# Supplementary material for: Role and mechanism of NCAPD3 in promoting malignant behaviors in gastric cancer
Source: Front Pharmacol. 2024 Apr 22;15:1341039. doi: 10.3389/fphar.2024.1341039 (PMC11070777; doi:10.3389/fphar.2024.1341039)
Supplement: Supplementary file 11 [file DataSheet2.ZIP › GSEA/GO_CC/my_analysis.Gsea.1599461408869/GO_NEURON_PROJECTION.html]

Details for gene set GO\_NEURON\_PROJECTION[GSEA]

|  || Dataset | filtered\_dataset.sample\_info.cls#WT\_versus\_NCAPD3\_MUT |
| Phenotype | sample\_info.cls#WT\_versus\_NCAPD3\_MUT |
| Upregulated in class | WT |
| GeneSet | GO\_NEURON\_PROJECTION |
| Enrichment Score (ES) | 0.17411773 |
| Normalized Enrichment Score (NES) | 1.1845474 |
| Nominal p-value | 0.22741432 |
| FDR q-value | 0.8632442 |
| FWER p-Value | 1.0 |
Table: GSEA Results Summary

  

Fig 1: Enrichment plot: GO\_NEURON\_PROJECTION      
 Profile of the Running ES Score & Positions of GeneSet Members on the Rank Ordered List

  

| SYMBOL | TITLE | RANK IN GENE LIST | RANK METRIC SCORE | RUNNING ES | CORE ENRICHMENT || 1 | 659 | BMPR2 | 16 | 1.047 | 0.0070 | Yes |
| 2 | 323 | APBB2 | 35 | 0.977 | 0.0112 | Yes |
| 3 | 222236 | NAPEPLD | 36 | 0.977 | 0.0291 | Yes |
| 4 | 23322 | RPGRIP1L | 60 | 0.871 | 0.0275 | Yes |
| 5 | 6421 | SFPQ | 87 | 0.818 | 0.0228 | Yes |
| 6 | 7976 | FZD3 | 88 | 0.815 | 0.0377 | Yes |
| 7 | 56288 | PARD3 | 99 | 0.790 | 0.0445 | Yes |
| 8 | 5967 | REG1A | 100 | 0.788 | 0.0589 | Yes |
| 9 | 56104 | PCDHGB1 | 115 | 0.781 | 0.0626 | Yes |
| 10 | 50861 | STMN3 | 126 | 0.777 | 0.0692 | Yes |
| 11 | 23380 | SRGAP2 | 142 | 0.757 | 0.0717 | Yes |
| 12 | 55714 | TENM3 | 149 | 0.736 | 0.0806 | Yes |
| 13 | 3770 | KCNJ14 | 157 | 0.721 | 0.0885 | Yes |
| 14 | 7248 | TSC1 | 158 | 0.718 | 0.1016 | Yes |
| 15 | 817 | CAMK2D | 165 | 0.710 | 0.1100 | Yes |
| 16 | 613 | BCR | 180 | 0.695 | 0.1121 | Yes |
| 17 | 54434 | SSH1 | 183 | 0.692 | 0.1232 | Yes |
| 18 | 9897 | KIAA0196 | 197 | 0.682 | 0.1258 | Yes |
| 19 | 9456 | HOMER1 | 198 | 0.680 | 0.1383 | Yes |
| 20 | 26278 | SACS | 205 | 0.669 | 0.1460 | Yes |
| 21 | 8831 | SYNGAP1 | 236 | 0.647 | 0.1350 | Yes |
| 22 | 57521 | RPTOR | 239 | 0.645 | 0.1453 | Yes |
| 23 | 23001 | WDFY3 | 240 | 0.642 | 0.1570 | Yes |
| 24 | 5701 | PSMC2 | 244 | 0.636 | 0.1664 | Yes |
| 25 | 55238 | SLC38A7 | 250 | 0.630 | 0.1741 | Yes |
| 26 | 5728 | PTEN | 285 | 0.606 | 0.1594 | No |
| 27 | 538 | ATP7A | 305 | 0.588 | 0.1558 | No |
| 28 | 7014 | TERF2 | 323 | 0.579 | 0.1534 | No |
| 29 | 6715 | SRD5A1 | 325 | 0.578 | 0.1633 | No |
| 30 | 9743 | ARHGAP32 | 413 | 0.528 | 0.1069 | No |
| 31 | 23242 | COBL | 426 | 0.516 | 0.1073 | No |
| 32 | 4771 | NF2 | 428 | 0.516 | 0.1160 | No |
| 33 | 6507 | SLC1A3 | 464 | 0.496 | 0.0985 | No |
| 34 | 34 | ACADM | 530 | 0.457 | 0.0576 | No |
| 35 | 8195 | MKKS | 542 | 0.449 | 0.0574 | No |
| 36 | 3836 | KPNA1 | 548 | 0.448 | 0.0618 | No |
| 37 | 5860 | QDPR | 597 | 0.425 | 0.0332 | No |
| 38 | 10445 | MCRS1 | 617 | 0.414 | 0.0264 | No |
| 39 | 10280 | SIGMAR1 | 655 | 0.399 | 0.0056 | No |
| 40 | 57679 | ALS2 | 656 | 0.399 | 0.0129 | No |
| 41 | 23064 | SETX | 657 | 0.398 | 0.0202 | No |
| 42 | 5190 | PEX6 | 694 | 0.381 | -0.0002 | No |
| 43 | 2932 | GSK3B | 714 | 0.367 | -0.0079 | No |
| 44 | 8772 | FADD | 742 | 0.355 | -0.0219 | No |
| 45 | 55646 | LYAR | 761 | 0.342 | -0.0293 | No |
| 46 | 23294 | ANKS1A | 768 | 0.339 | -0.0276 | No |
| 47 | 23767 | FLRT3 | 781 | 0.333 | -0.0306 | No |
| 48 | 1605 | DAG1 | 823 | 0.304 | -0.0561 | No |
| 49 | 3312 | HSPA8 | 848 | 0.244 | -0.0699 | No |
| 50 | 3673 | ITGA2 | 858 | -0.264 | -0.0719 | No |
| 51 | 5723 | PSPH | 859 | -0.267 | -0.0670 | No |
| 52 | 55450 | CAMK2N1 | 861 | -0.269 | -0.0628 | No |
| 53 | 667 | DST | 886 | -0.303 | -0.0755 | No |
| 54 | 9181 | ARHGEF2 | 902 | -0.326 | -0.0809 | No |
| 55 | 23710 | GABARAPL1 | 913 | -0.341 | -0.0823 | No |
| 56 | 2317 | FLNB | 936 | -0.360 | -0.0924 | No |
| 57 | 81544 | GDPD5 | 961 | -0.381 | -0.1036 | No |
| 58 | 5621 | PRNP | 970 | -0.387 | -0.1026 | No |
| 59 | 55591 | VEZT | 973 | -0.389 | -0.0970 | No |
| 60 | 9572 | NR1D1 | 977 | -0.392 | -0.0921 | No |
| 61 | 3176 | HNMT | 991 | -0.398 | -0.0947 | No |
| 62 | 23046 | KIF21B | 1034 | -0.433 | -0.1186 | No |
| 63 | 80315 | CPEB4 | 1053 | -0.448 | -0.1241 | No |
| 64 | 1191 | CLU | 1056 | -0.450 | -0.1174 | No |
| 65 | 5818 | PVRL1 | 1073 | -0.458 | -0.1211 | No |
| 66 | 283 | ANG | 1074 | -0.458 | -0.1128 | No |
| 67 | 9939 | RBM8A | 1093 | -0.474 | -0.1177 | No |
| 68 | 26053 | AUTS2 | 1113 | -0.485 | -0.1233 | No |
| 69 | 1263 | PLK3 | 1134 | -0.502 | -0.1293 | No |
| 70 | 7074 | TIAM1 | 1143 | -0.508 | -0.1260 | No |
| 71 | 3706 | ITPKA | 1150 | -0.514 | -0.1212 | No |
| 72 | 54407 | SLC38A2 | 1182 | -0.553 | -0.1346 | No |
| 73 | 4651 | MYO10 | 1213 | -0.588 | -0.1466 | No |
| 74 | 8061 | FOSL1 | 1222 | -0.597 | -0.1417 | No |
| 75 | 2043 | EPHA4 | 1228 | -0.602 | -0.1345 | No |
| 76 | 26999 | CYFIP2 | 1234 | -0.611 | -0.1271 | No |
| 77 | 8408 | ULK1 | 1243 | -0.622 | -0.1218 | No |
| 78 | 55638 | SYBU | 1275 | -0.663 | -0.1332 | No |
| 79 | 286 | ANK1 | 1284 | -0.672 | -0.1270 | No |
| 80 | 54800 | KLHL24 | 1287 | -0.677 | -0.1161 | No |
| 81 | 54464 | XRN1 | 1301 | -0.703 | -0.1131 | No |
| 82 | 885 | CCK | 1302 | -0.703 | -0.1003 | No |
| 83 | 3084 | NRG1 | 1320 | -0.739 | -0.0997 | No |
| 84 | 6509 | SLC1A4 | 1330 | -0.752 | -0.0928 | No |
| 85 | 10611 | PDLIM5 | 1335 | -0.758 | -0.0819 | No |
| 86 | 5997 | RGS2 | 1336 | -0.770 | -0.0679 | No |
| 87 | 4734 | NEDD4 | 1369 | -0.856 | -0.0765 | No |
| 88 | 6711 | SPTBN1 | 1381 | -0.925 | -0.0679 | No |
| 89 | 4919 | ROR1 | 1385 | -0.954 | -0.0528 | No |
| 90 | 8867 | SYNJ1 | 1387 | -0.979 | -0.0356 | No |
| 91 | 493 | ATP2B4 | 1401 | -1.166 | -0.0242 | No |
| 92 | 760 | CA2 | 1410 | -1.654 | -0.0000 | No |
Table: GSEA details [plain text format]

  

Fig 2: GO\_NEURON\_PROJECTION      
 Blue-Pink O' Gram in the Space of the Analyzed GeneSet

  

Fig 3: GO\_NEURON\_PROJECTION: Random ES distribution      
 Gene set null distribution of ES for **GO\_NEURON\_PROJECTION**
